# This Class Definitely Changed My Opinion of Chemistry: How a Pedagogical Course Reform Improved Students’ Chemistry Attitudes

**DOI:** 10.1021/acsomega.5c02931

**Published:** 2025-07-30

**Authors:** Nicole M. James, Kodinna Anachebe, Nicole D. LaDue

**Affiliations:** † Chemistry Department, 6686 Reed College, 3203 SE Woodstock Blvd, Portland, Oregon 97202, United States; ‡ Department of Earth, Atmosphere and Environment, 2848 Northern Illinois University, DeKalb, Illinois 60115, United States

## Abstract

Introductory chemistry is a gateway course that influences students’ persistence in science-related degree programs and careers. Here, we investigate how a deliberate practice-informed introductory chemistry reform influenced student attitudes toward chemistry. Through open coding and thematic analysis of 5 focus group discussions, we illustrate causal mechanisms between course attributes and students’ affective outcomes and attitudes toward chemistry. The use of student-centered instructional practices and frequent opportunities for students to practice and receive feedback are consistently described to prompt positive affective outcomes, while the lack of these practices and opportunities is described to prompt negative affective outcomes. Participants directly indicate that these attributes of the course influenced their broader attitudes toward chemistry as a discipline. For educators who view this to be transferable to their context, these findings illustrate ways to incorporate deliberate practice tenants of motivation, practice, and feedback to positively change students’ attitudes toward chemistry.

## Graphical abstract

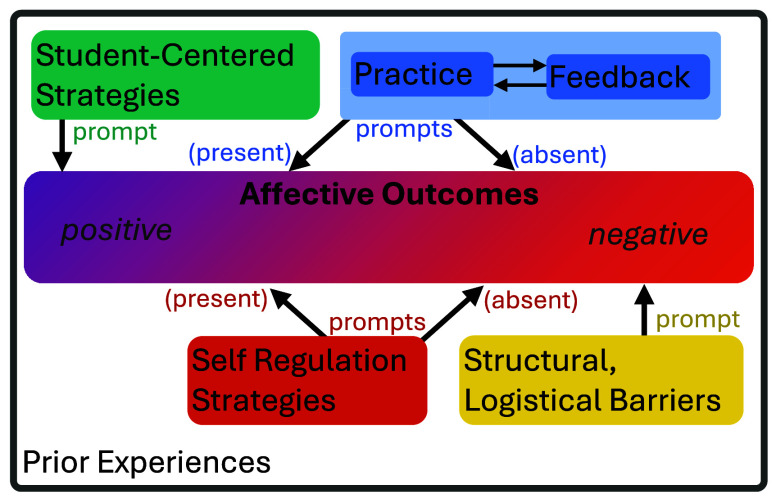

## Introduction

It is well documented that chemistry often serves as a gatekeeper, restricting students’ ability to complete degree programs in science, engineering, and medicine.^1,2^ This persists today despite sustained efforts to develop pedagogical practices and curricula that better support student chemistry learning.^3–5^ For example, there have been significant efforts to develop and implement “active learning”-oriented practices such as process-oriented guided-inquiry learning,^6^ problem-based learning,^7^ flipped instruction,^8^ and reformed curricula.^9,10^ However, adoption of such practices has been relatively slow, particularly in chemistry.^4,5^ Recent studies have tied this to how personal and institutional factors influence educators’ teaching practices.^3,11–14^

Furthermore, while evidence-based pedagogical practices have been shown to consistently positively impact student learning outcomes, the extent of their impact is highly variable. Freeman et al. performed a meta-analysis of 225 active learning intervention studies and found a consistent reduction in the failure rate associated with active learning instruction compared to ‘traditional’ lecture.^15^ On average, this reflected a failure rate reduction from 33.8% (traditional lecture) to 21.8% (active learning). However, the distribution was broad: the percent change in the failure rate ranged from approximately −10% to 50%.^15^ Subsequently, Rahman and Lewis performed a meta-analysis comparing the relative effectiveness of different active learning practices.^16^ They similarly found that the practices generally improved student learning outcomes, but recommended anticipating effect sizes associated with such practices to range from 0.29 to 0.62. Rahman and Lewis highlight that this variability may be a result of confounding variables that could not be accounted for with the available data, such as course size and other local contextual factors. Consistent with this, close examinations of flipped learning practices have shown generally positive but variable student outcomes.^17–19^ In light of this, there is a need to not only characterize associations between course reform efforts and student outcomes, but to investigate the causal mechanisms through which the reform influences those outcomes. Improved understanding of these causal mechanisms would better allow educators to select, implement, and adapt course practices based on their context’s needs, while maximizing effectiveness.

We have previously shared our design and implementation of a pedagogical reform in an introductory chemistry course and its association with student outcomes.^20^ This reform was informed by the deliberate practice framework for learning,^21,22^ with our thinking also influenced by overlapping social constructivist learning frameworks such as self-regulated learning^23,24^ and social cognitive career theory.^25,26^ In our view, deliberate practice and many other social constructivist frameworks all acknowledge the role student effort plays in constructing knowledge. As a result, student motivation to put forth effort is also relevant to their learning. Theoretical frameworks for student motivation, such as self-determination theory,^27^ expectancy-value theory,^28^ and others,^29–31^ consistently tie students’ motivation to their thinking, perceptions, and experiences. In light of this, our prior study^20^ sought to complement our discussion of student content learning with a characterization of students’ attitudes toward chemistry.

We use a broad conception of attitudes, defined as a tendency of favorable or unfavorable evaluative responses about an entity (or “attitude object”).^32^ “Attitudes” thus can be considered to encapsulate many constructs, such as beliefs, interests, values, self-efficacy, and more.^33^ As a whole, positive attitudes toward chemistry have been associated with students’ aspirations to pursue chemistry^34^ and students’ achievement in chemistry.^35–37^ While student attitudes tend to vary across scientific disciplines,^38^ the general association between positive attitudes and greater disciplinary achievement has been observed widely in STEM.^39–43^ In chemistry, student attitudes have been seen to vary along demographic lines,^44^ and to be impacted by course structure.^44–46^ Given the robust connection between student attitudes and their broader outcomes, insight into the nature of how attitudinal changes are influenced by course structure is useful for instructional design.

As previously reported in our assessment of an introductory chemistry pedagogical reform,^20^ we characterized student attitudes via a quantitative pre- and post-survey that included the revised Attitudes toward the Study of Chemistry Inventory.^47^ We did not have a preconceived expectation of how student attitudes might be influenced by the course reform, but we were nonetheless surprised: the reformed course was associated with a statistically significant change toward more positively oriented student attitudes, while the unreformed courses were both associated with a decisive change to more negatively oriented attitudes.^20^ However, the data collected did not provide insight into the mechanisms that caused these changes. In this article, we report a follow-up study we conducted to identify and describe the casual mechanisms that underlie these attitudinal changes.

Through analysis of focus group data, we aim to answer the following research questions:

1. How do students explain the relationship between the design of their introductory chemistry course and their experience with chemistry?
2. Through what mechanisms did the reformed course result in different student attitudes toward chemistry than the unreformed courses?

## Methods

### Theoretical Foundations

In this study, we employed a critical realist ontology.^48^ That is: we acknowledge there exist complex causal relationships that inform reality, which are influenced by personal factors and societal systems. Consequently, we expected to find a varied landscape of multifaceted tendencies and sought to understand general themes about these tendencies. This view also prompted us to employ a contextualist epistemology,^49^ which asserts that one’s context directly influences how one experiences reality, making it impossible to isolate one’s experience from its context. This impacts our study design, for example, by motivating the use of qualitative methods that capture rich detail about a variety of experiences. It also impacts our analysis, for example by prompting us to consider how apparent contradictions in participants’ experience may be mutually consistent when viewed in their larger context. In other words: our ontological and epistemology foundations prompted us to look for general tendencies and themes among participants’ experiences, considered within their individual context.

In alignment with these foundations, we employ an antideficit frame^50^ in our consideration of students and instructors. We operate under the assumption that students want to and can be successful learners and that instructors want to and can be effective teachers, but the systems and structures they operate within can complicate achieving these goals. This influences our analysis, for example, by prompting us to believe that students and instructors possess the qualities necessary to achieve their goals. This can indicate a need to examine ways that perceived deficiencies may not be a result of individuals’ inherent abilities, but of limitations in the systems and structures they operate within.

Our conceptualization of attitudes is guided by multiple theories and frameworks. Attitudes are often operationalized through a tripartite or neotripartite model, where an individual’s attitudes are latent and manifest through behavioral, cognitive, and/or affective mechanisms.^32,51^ The neotripartite model more explicitly acknowledges that behavioral, cognitive, and affective measures are interrelated and may not be separable.^32,52^ However, attitudes have also been operationalized through expectancy-value models,^53–55^ where one’s attitude is informed by their perception of the likelihood of particular outcomes, and their evaluation of those outcomes. (Neo-)­tripartite and expectancy-value models generally point toward distinct approaches for designing quantitative instruments to measure attitudes.^56–58^ However, for our qualitative analysis, we view these models to be mutually compatible. We perceive (neo-)­tripartite models to prompt us to attend to the affective, cognitive, and behavioral dimensions that attitudes manifest in. Simultaneously, expectancy-value models prompt us to consider how affective and cognitive judgements impact engagement in particular behaviors. In this way, we view both models as distinct, but complementary frameworks to guide our thinking. We also acknowledge that both models overlap with other frameworks and theories, such as for motivation^30^ and self-efficacy,^59^ which we also find useful for guiding our thinking.

Collectively, we view these frameworks to provide a complementary and mutually compatible structure for our qualitative interpretations. For example: a participant may describe enjoying attending class because they realized it was possible for them to learn chemistry, and they are now more interested in continuing to learn chemistry. Self-efficacy, motivation, (neo)­tripartite, or expectancy-value frameworks would each prompt us to interpret this statement slightly differently, but the general conclusion with respect to our research questions would be the same: the course created an environment where the participant felt more confident and thought they were able to succeed, which positively impacted their attitude toward chemistry. Thus, for the purposes of this study we conceptualize attitudes broadly and acknowledge that these multiple overlapping theories collectively guide our interpretations.

In addition, the deliberate practice framework that informed the structure of the reformed course section also guides our analysis of how the course may have transformed student attitudes. Deliberate practice^21,22^ proposes that students learn by engaging in effortful practice, accompanied by feedback that informs subsequent practice. By nature of being effortful, this practice is tiring; deliberate practice thus suggests that practice is best accomplished in short (1–2 h) but frequent sessions and relies upon students’ motivation to engage. In designing focus group questions, we intentionally avoided “leading” participants toward a discussion of these tenants. However, in our analysis, this framework prompts us to attend to areas where participants’ comments align with these tenants of practice, feedback, and motivation.

### Study Setting and Context

This study received approval from the Northern Illinois University Institutional Review Board (IRB #HS20-0052). Participants provided written consent to participate at the time of data collection. These data were collected at a public, high research activity (R2) university in the United States. This institution has an enrollment of approximately 16,000 students, composed of an appreciable combination of residential, commuter, full-time, and part-time students with varied racial and socioeconomic backgrounds. Here we investigate the attitudes of students enrolled in a one-semester introductory chemistry course in Fall 2019. In Fall 2019, one section was subject to a pedagogical reform (Section R); two concurrent, unreformed sections (Sections UA and UB) were used as comparator groups.

### Course Context: Structure and Learning Outcomes

A prior publication^20^ reports characterization of course instruction and student outcomes using course observations, course syllabi, student pre/post surveys. Our aim here is to focus on the unique understanding that can be gained from focus groups of students who had been enrolled in these courses. However, the course structures and student outcomes are important context for these data. Thus, we here summarize these aspects, and refer to our prior publication^20^ for greater detail.

This one-semester introductory chemistry course provides an overview of topics from the full year of general chemistry; it serves as a preparatory course for students who will subsequently take general chemistry, but it can also be taken on its own to satisfy requirements for multiple degree programs in areas such as health and engineering. The department establishes requirements for the course text and grading scheme. Course sections range from approximately 80–150 students (see Table 1), and met weekly for either three 50 min lectures (Sections UA, R) or two 80 min lectures (Section UB). Observations using the Course Observation Protocol for Undergraduate STEM^60^ indicated that the method of instruction was predominantly didactic lecture in the unreformed sections (UA and UB), and interactive lecture with frequent short group work in the reformed section.^20^ All course sections had two forms of regular homework: problem sets and reading assignments. Problem sets were assigned via the publisher’s online homework platform, involving publisher-developed questions. An analysis of exam items indicated that all courses primarily assessed recall and algorithmic problem solving, with the reformed course (Section R) assessing slightly more “higher order” skills such as evaluation and transformation of chemical representations.^20^

**Table 1:** Course Enrollments and Number of Focus Group (FG) Participants

**course**	**course enrollment**	**FG**	**FG participants (** *N* **)**
R	121	R-1	7
		R-2	5
UA	153	UA-1	8
		UA-2	6
UB	79	UB-1	5

Instructors of the unreformed course sections (UA, UB) consented to participate in this study, making their courses available for data collection. They were asked to simply teach however they typically would, in a “business as usual” sense. The reformed course section incorporated deliberate practice tenants through multiple avenues.^20^ For example, the course aimed to promote motivation through pedagogical transparency and explicitly connecting the content to students’ real-world experiences and academic goals. Practice and feedback were structurally incorporated, for example, through pre-class assignments, in-class active practice, and homework assignments. The deliberate practice framework emphasizes the importance of spacing effortful practice out over time, which prompted breaking the weekly reading and homework assignments up into smaller components due three times per week.

Student course outcomes were assessed through pre/post surveys, assignment performance, and course grades. Students in the reformed course section performed higher on shared exam items and earned statistically higher overall course grades, with a medium to medium-large effect size.^20^ Consequently, the reformed section had a lower rate of nonpassing (D, W, F) grades.^61^

## Data Collection

All students who had been enrolled in Sections R, UA, and UB during Fall 2019 were invited via email to participate in focus group discussions about their experience in these courses. Food was provided and each participant was compensated with $20 at the end of the discussion. A geology education research graduate student (Erika Zocher), who had experience conducting interviews and focus groups, served as the primary facilitator for all focus groups. All focus groups had a secondary facilitator: either author N.M.J. or a geology education research graduate student (Bailey Kreager). Focus groups were audio and video recorded; recordings were transcribed using a transcription service. Because author N.M.J. was the instructor of Section R, author N.D.L. coordinated recruitment for focus groups involving Section R. N.M.J. was not present for these focus groups and does not have access to identifiable information about the participants from Section R.

Five focus groups were conducted in January and February 2020, after Fall 2019 course grades were posted. As shown in Table 1, focus group sizes ranged from 5 to 8 participants.

Based on our theoretical foundations, we found it relevant to examine the similarity between the course sections’ focus group participants, particularly in terms of career plans and/or course grades. Thus, participants were asked to self-report these characteristics through an optional form at the end of the focus group (Figure 1).

**Figure 1:**
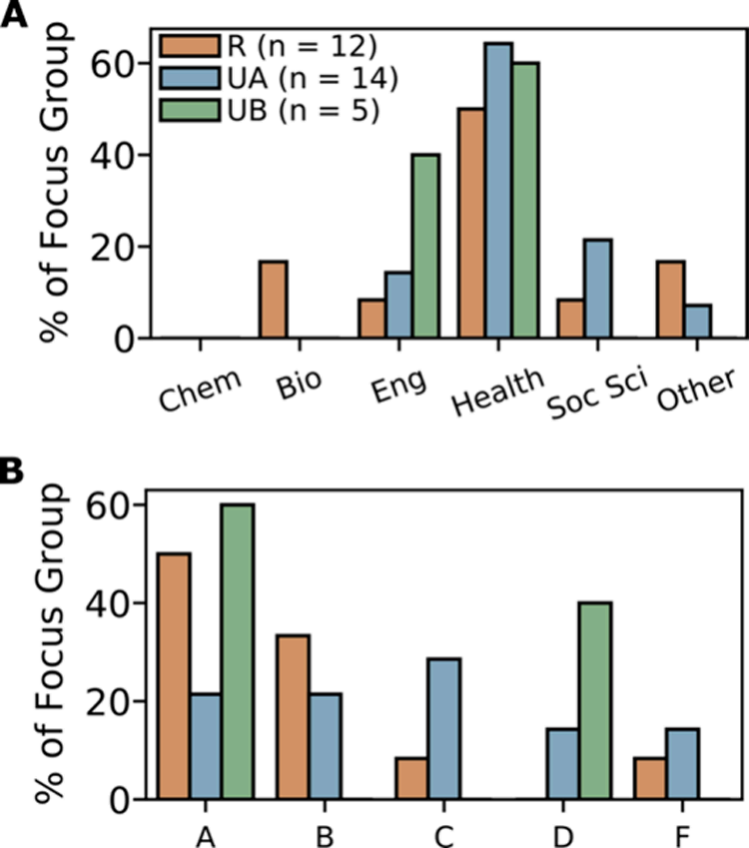
Focus group participants’ self-reported intended degree program (A) and introductory chemistry course grade (B), as a function of course section. Intended degree program options included: “Chemistry/Biochemistry” (Chem), “Biology” (Bio), “Engineering-related” (Eng), “Health-related (e.g., nursing, kinesiology, nutrition)” (Health), “Social science-related” (Soc Sci), and “Other” (Other). Letter grades were assigned with ± designations, but have been combined (i.e., “A” reflects the total number of A+, A, and A– grades).

As this is a qualitative study, we are not concerned with the exact rates of focus group participants’ grades or intended degree programs. We seek to capture varied experiences, and are not quantifying these experiences. We use this data as a proxy for assessing how likely it is that varied experiences are captured from each of the course sections. For each section, participants represented a variety of career paths, with most students pursuing health-related programs and no students pursuing a chemistry or biochemistry degree. Similarly, participants from each section received a variety of course grades, including both passing (A, B, C) and nonpassing (D, F) grades.

All focus groups began with introductions and an ice-breaker activity. For each question, participants were given a short time to collect their thoughts before discussion. Paper and pens were provided so participants could jot notes if they found it helpful. The primary interviewer facilitated the discussion, intentionally made space for contributions from a variety of participants, framed the discussion to normalize sharing differing or conflicting experiences, and encouraged participants to share even if they felt they were repeating something that had already been said. Since there is a significant cognitive load associated with running the session, the secondary interviewer’s focus was to actively listen, be attentive to group dynamics, and ask probing questions as needed to clarify comments or elicit broader participation. Focus groups were semi-structured; core questions are provided in Table 2.

**Table 2:** Focus Group Discussion Prompts

**target or purpose**	**discussion question or prompt**
warm-up	How did [course] fit into your academic goals?
attitudes toward chemistry	Before you ever set foot in [course], what did you think about chemistry as a subject?
	Did your experiences in the course change your perceptions of chemistry?
	(common follow-ups: “Can anyone provide a specific example or experience?”; “What was it about the course that changed your perception?”)
	Describe how you might change the course to improve your experience with chemistry
self-efficacy in chemistry	How would you describe your confidence before and after taking [course]?
	Tell me about a time when you felt you were able to learn in [course]
	What types of strategies or resources did you use to support your own success in [course]?
	Tell me about a time when something felt especially challenging in [course]
conclusion/wrap-up	Is there anything else you’d like to add about your experience in [course] that has not been sufficiently covered yet in our conversation?

## Data Analysis

In 2022–2023, author K.A. began analyzing the collected focus group data as part of their undergraduate senior thesis. K.A. watched recordings of all focus groups and corrected any errors in the digital transcripts. Analysis occurred in two main stages: open coding, followed by thematic analysis.

### Open Coding

K.A. inductively open-coded two transcripts (UA-1, UB) by annotating physical copies of the transcripts. N.M.J. independently reviewed these transcripts and met with K.A. to discuss K.A.’s open codes to consensus. This process developed the initial codebook. Using this codebook, K.A. then recoded these same transcripts using a qualitative analysis software program (MaxQDA). The remaining transcripts were then coded in MaxQDA; after coding each additional transcript, take-aways and revisions or additions to codes were discussed to consensus with N.M.J. When revisions to the codebook were made, previously coded interviews were recoded to align with the revised codebook. At all stages of the analysis, K.A. wrote analytical memos to document their process and reasoning.

### Thematic Analysis

K.A. and N.M.J. collaboratively identified descriptive themes in the data. To do so, each code was written on an individual piece of paper and placed on a surface where all codes were visible. K.A. initiated theme generation by discussing key features and aspects they noticed in and across the focus group transcripts, and discussed this collaboratively with N.M.J. Through this process, K.A. and N.M.J. grouped codes into thematic groups by consensus. The characteristics of each theme, and the relationships between each theme, were developed through consensus discussions. This resulted in the development of a thematic map. N.M.J. later re-examined all focus group transcripts for disconfirming evidence, and found none.

### Trustworthiness

We attended to trustworthiness criteria of transferability, credibility, dependability, and confirmability.^62,63^ Transferability is supported through detailed descriptions of the courses investigated in both this article and our prior publication.^20^ Credibility was supported through consensus discussions between researchers and triangulating codes and themes between participants within a focus group, between focus groups, and between course sections. Credibility was also supported through extended engagement with the data, such as watching recordings, recoding transcripts, and examining transcripts for disconfirming evidence. Dependability was supported by documenting data collection methods (e.g., focus group protocol) and the methods and reasoning that informed data analysis (e.g., analytical memos). Confirmability was attended to through researcher reflexivity and consistency with evidence-based theoretical frameworks. To make our reflexivity more visible to the reader, at the beginning of this section we have included explicit discussion of the ontological and epistemological assumptions that informed our work, and how our use of theoretical frameworks guided our thinking.

## Results

The results presented here were developed through the analysis of all focus group discussions, and thus all themes are fundamentally grounded in the experiences of participants in all sections (UA, UB, R). By looking for similarities and difference within and between each section, we can develop a fuller picture of the possible causes of attitudinal changes, addressing Research Question 1. This causal mechanistic understanding enables an evidence-based discussion of how the course design influenced attitudinal changes such as if and how the reformed section prompted positive changes, addressing Research Question 2.

Descriptive themes include *Affective Outcomes, Feedback, Logistical and Structural Barriers, Self-Regulation Strategies, Student-Centered Strategies, Practice,* and *Prior Experiences*. In the Supporting Information, we provide the codebook and indicate which codes motivated the generation of each theme. We visually represent these themes, and the relationships between them, with a thematic map (Figure 2). Importantly, this map represents a “landscape” of possible paths an individual’s experience could take, informed by the experiences of participants from all sections. Themes may manifest differently in different sections, or even for different participants in the same section. Here we first focus on unpacking the evidence for each theme. Where relevant, we note when certain experiences appear more or less prevalent in a specific course section, but in the Discussion we more directly discuss how course design features impact the relative prevalence of these themes in the reformed vs. unreformed sections. For clarity, in-text references to theme names are capitalized and italicized throughout.

**Figure 2:**
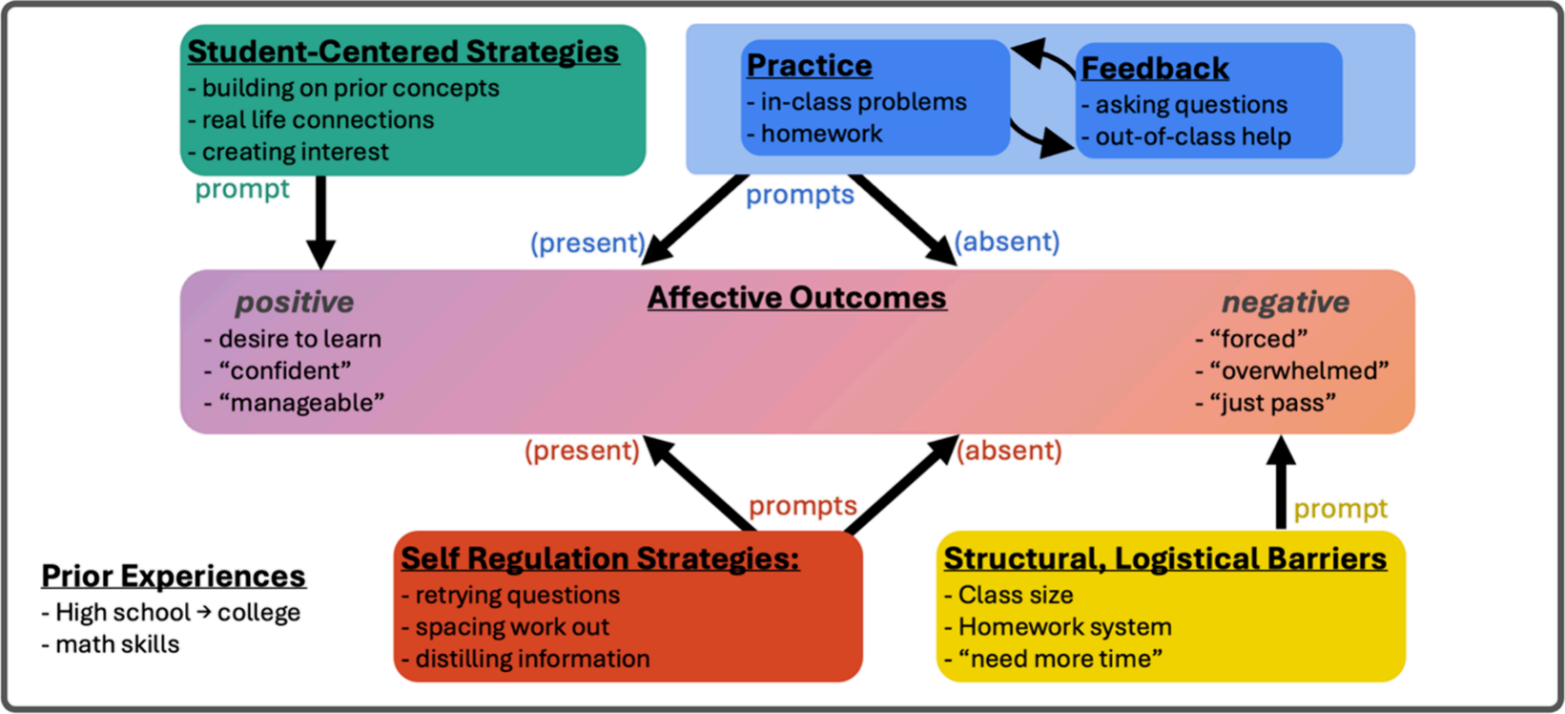
Map of themes generated through the analysis of all focus group discussions. Theme names are bolded and underlined. Example codes are provided to illustrate each theme; quotation marks indicate *in-vivo* codes.

### Affective Outcomes

The *Affective Outcomes* theme describes students’ self-reported thoughts and feelings about the course and chemistry as a discipline. We depict this theme as a spectrum to reflect how each individual experiences a variety of affective states, which collectively contribute to their overall affective outcome. In other words: we observe no “purely” positive or “purely” negative overall experiences, but rather combinations of experiences that, taken together, may be positively or negatively oriented to differing degrees.

Participants from all course sections describe instances of overwhelmed, isolated, and unable to learn:

“I mean, I was a freshman. I don’t know about you guys, but it’s my first semester at college, plus being in a sorority first semester and trying to join every club I can and managing homework and managing classes is really overwhelming, especially when you’re living by yourself.” (R‑2)

“[in this class] there’s a lot of just, ‘get through it. Don’t learn it. Do what you need to do to pass’ kind of thing.” (UA‑1)

“I hate chemistry with all my heart. I never want to take it again. And I think it’s more of the math in it is what I struggle in because I’m not really good at math.” (UA‑1)

“there’s less student-to-student connection because I didn’t really get to know--I sat by the same couple of people and we’d exchange a few words, but I never went to a study group. I feel my smaller classes where I’m sitting next to people, I could ask them for their number or ask them what they did with their work and stuff like that. It’s just more intimidating having a bigger class and not being able to feel like you could have a student-to-student connection.” (UA‑2)

“I tried to reach out and get help [···] for one of the assignments, and they told me to just google it, and pretty much turned me away. So that kind of discouraged me” (UB)

While negative experiences such as these are mentioned in all course sections, they are talked about in more depth and met with more agreement from other participants in the unreformed course section focus groups. Consequently, we perceive them to be more prevalent in the unreformed section focus groups than in the reformed section focus groups. Similarly, participants from all course sections describe instances where they felt able to learn, interested in learning, and connected with others, which we interpret to be positive experiences. However, these experiences are more prevalent among participants from the reformed course. For example:

“I’m probably not going to be taking any additional chemistry classes. I wish I kind of was [···] But yeah, I think it just made everything-- kind of made me think, “Okay, everything is doable,” I guess.” (R‑1)

“I like the classes where the teacher makes me want to come to class and loves it, and that’s why I loved chemistry because I wanted to come and I wanted to learn more.” (R‑2)

“she was so nice, so personable, answered any question, like if she was in the middle of talking and you would raise your hand, she’d stop and ask, which was really nice. So it really helped me learn.” (R‑2)

“I feel like [instructor’s] enthusiasm really hit me. Yeah, it definitely caught my eye and it just made me more awake, I guess [···] she was very enthusiastic and you could tell that she really loved her subject.” (UA‑2)

“[supplemental instructor] was like, so good that before I didn’t really like chem, but now like I love it, [···] next semester, my sophomore year, [I] really want to do tutoring for it. [···] I definitely feel like having that experience with her, like, allowed me to better understand the class itself.” (UB)

In many of these examples, we notice participants drawing a causal tie between course-based affective experiences and their broader attitudes toward chemistry as a discipline. For example:

“Yeah, [the instructor] changed my perception of [chemistry] because like I said earlier, I didn’t like it at all. Now, it’s not that--I don’t love chemistry, I’m like neutral with it because I don’t have a problem with it.” (R‑1)

“I think my perspective [on chemistry] did change. I used to think I should be an art major [···] But [this course] helped me know that understanding things is really satisfying, in the field of science. [···] I learned that I was capable of learning in the science field.” (R‑2)

“[before this course] I really hated science. [···] [but] after just taking the course it just boosted my confidence a lot and I’m ready to take the next level and all that. I like it now.” (UA‑2)

“[this course] literally made me change my major [···] like normally, I’m able to like overcome learning certain things even if it’s difficult, but I just felt like I was not retaining the information at all. [···] all my life, I wanted to be a forensic scientist and [this class] made me change my major and I did not want to do that anymore. [···] the job I’m trying to do, I need to know this but I was like—I don’t even love it anymore. I completely like, started hating chemistry.” (UB)

In this way, participants describe the course adjusting their attitudes toward chemistry positively or negatively. To answer our research questions, we are particularly interested in what course factors differentially push participants’ attitudes toward the positively- or negatively-oriented ‘sides’ of the *Affective Outcomes* spectrum, which we examine in the following themes.

### Prior Experiences

The *Prior Experiences* theme reflects instances where participants highlight the influence of past experiences on their course experience and/or chemistry attitudes. In many cases, this stems from prior chemistry courses, or the lack thereof:

“I had a poor experience when I was first introduced to chemistry in high school. I had a very bad teacher who didn’t explain the material in the fashion the whole class could understand. And so I came into [this class] a little bit wary” (R‑1)

“I needed [this course] for my major, so it was a requirement for me. I had a difficult time because I didn’t take chemistry in high school” (R‑2)

“I liked [chemistry] in high school a lot. More so because my teacher made me end up liking it [···] when I got the hang of it I was like, I like this” (UA‑2)

However, some participants also noted the relevancy of other past course experiences:

“physics helped me a lot for the laws part of chem. That was so easy for me because of physics. That doesn’t really make sense, but it was something I used and it helped a lot.” (UA‑1)

or their experience transitioning from high school to college more generally:

“the second exam I failed and it was like—oh. Okay now. It’s not [···] high school, I took AP classes and like kind of skimmed through high school without really studying” (UB).

Notably, the prior experiences that participants remark on are all course-based academic experiences, primarily from high school. Participants did not comment on the relevancy of nonacademic lived experiences or cultural knowledge as assets to draw upon in the course or in their understanding of chemistry. In this way, participants’ prior experiences generally seem to position chemistry and chemistry knowledge as “separate from” their lives in general, connected to them through its status as a prerequisite for their intended career paths.

This theme impacts how all other themes manifest, and thus we depict it in Figure 2 as a box surrounding all other descriptive themes. It informs participants’ ‘starting’ affective states at the beginning of the course, which are then influenced by their course experience. As discussed more in the following themes, these prior experiences also influence how participants describe being able or unable to overcome challenges they encountered in the course.

### Practice and Feedback

The *Practice* theme reflects participants’ descriptions of times they engaged in practice and/or opportunities they wish they had for practice. The *Feedback* theme reflects participants’ descriptions of receiving or struggling to receive feedback on their learning. We discuss these themes together and indicate them as a unit in Figure 2 because they are tightly coupled in participants’ descriptions. For example, participants often remarked that opportunities to practice and receive feedback have a positive impact on their learning and affective outcomes:

“I really enjoyed that if she saw-- she would take [an audience response] question and if people had problems with it or if she saw people struggled with something on the test, she would post something on [the course LMS], and that’d be even more helpful” (R‑1)

“It was also really helpful that on the homework it was basically once you put the answer down, you could check your answer to make sure it’s good, so if it is, then you can move on, and if not, then you’re like, ‘Okay, I need to revisit my notes’.” (R‑2)

In contrast, practice opportunities not accompanied with such feedback do not appear associated with positive student perceptions. This arises in multiple areas of focus group discussions; one illustrative case involves course reading assignments. In the unreformed sections, reading assignments were due weekly and included adaptive comprehension questions. If an answer was incorrect, additional versions of the question were provided, and it was only possible to proceed to the next section after completing the question successfully. Many participants described these reading assignments in a negative light:

“[···] the reading questions, it could go up to like 80 questions and if you get it wrong, you got to keep repeating it. It won’t go down. I hated that so after a while I just got sick of it, started clicking things, and I just got the right answers. [···] I just don’t think the reading questions were even helpful” (UA‑1)

“It was specifically the [reading assignments] that were bad [···] you have to master 70 questions, and if you got it wrong you were stuck at that number until you got a question right, so I would spend like 2 h sometimes” (UB)

However, in a few cases, participants describe their experience in a more neutral or positive light:

“I was kind of neutral on it [···] I liked the fact that with the [reading assignments] [···] you knew that you had gotten everything right at the end, so at least for me it was like, I can be sure that I figured it out, and yeah—most of the [reading assignment] stuff wasn’t on the test, but like, you got all the basic concept stuff [···] it helped me understanding what I was doing more on the test.” (UB)

“Even though the [reading assignments] were long, I actually used the [reading assignments] to take notes instead of doing notes in class.” (UA‑2).

Participants who described the reading assignments in a negative light did not perceive the reading responses to provide feedback helpful for their learning; rather, when they got questions wrong, they often described guessing or disengaging with the assignment. In contrast, participants who perceived the reading assignments in a more neutral or positive light tended to describe ways the effort they put into the reading assignments did contribute to their learning. This highlights how one practice-feedback opportunity may not “work” equally for all students. We can glean some insight into possible factors that cause this variation through one participant’s description of their reading assignment experience:

“The homework, first of all, was very overwhelming. We had 75 questions for the reading [···] It probably took me like four or five days to do-- probably a week just because I didn’t want to do it all at once. And then there was this one time when I did do it all at once and it took me like six hours to do. It was a lot to do and I feel like half the time, a lot of people probably don’t even read the reading. They probably just go through and click the answers [···] I did that for a while and I didn’t learn anything, so I had to stop doing that” (UA‑1)

This participant was able to work through the emotional impact of how “overwhelming” they found the practice-feedback opportunity and make their effortful engagement more feasible by spacing out the work. They also recognized that not all ways of engaging with the practice-feedback opportunity benefitted their learning, and they were able to adapt their behavior to engage in ways that did benefit their learning. Not all participants successfully navigated this, and time limitations due to other responsibilities appear to play a role:

“I had [reading assignment] in like two other classes [···] it was like, just killing me. [···] all three of them had [reading assignments] and some of them were like the 70, 80 questions each, so I was at it for like hours, so I kind of just stopped doing it because I got sick of having to do it for all my classes” (UB)

In addition to variations in the time pressure experienced by participants, it is also possible that variations in prior experiences—and the prior knowledge gained from those experiences—creates differences in how feasible it is for students to understand and use the reading assignment feedback. We see this reflected above in the metacognitive skills required to find ways to engage effectively with the assignment. Additionally, this may relate to how the inability to “skip” a section is a common source of frustration:

“if you’re really struggling with the concepts, sometimes it can help but sometimes it can also be more frustrating, like, instead of just being able to skip it like, ‘okay I just don’t understand it right now let me skip it,’ it will just keep—no you have to do it, you have to do it, you have to do it.” (UB)

We suspect that participants whose prior experiences provided them with more foundational chemistry knowledge may be less likely to encounter areas where the reading assignment does not provide sufficient feedback to successfully complete the section. However, if additional feedback is needed, the inability to “skip” a section could obstruct the use of strategies to compensate. For example, it prevents the student from identifying all areas they struggled in at once and seeking out help in those areas at tutoring or office hours.

The reformed course section also involved a reading assignment, but it did not use the same platform and was not adaptive. Prior to each class, students read a section of the textbook and/or watched an online video and responded to 3–5 short answer questions. Participants’ comments about this reading assignment were very different:

“So everyday before class, we would have a reading response due, and it would be on what we would do in class. So we would go in class knowing what we would learn about, so like that we can have questions ahead of time if any” (R‑1)

“[she would] always have something ready to explain because she’d always know what people are usually having trouble with based on test results or reading responses. She’d know what to help people on. So those were nice” (R‑1).

Here, participants conveyed a generally neutral-to-positive perception of the reading responses, and appear to recognize areas where the reading assignments allow them useful feedback. However, there is still a suggestion that prior experience and/or foundational knowledge may play a role in participants’ experience of the assignment:

“[···] with the reading responses, it was kind of hard because like, even if you did read the book, and like you’d go to answer them, sometimes you couldn’t find the spot that the reading responses were leading towards. So even as you read the book, and then you look at the questions, you’re like, ‘I don’t know what this is’.” (R‑2)

However, this participant frames this as a relatively minor point—it does not appear to prompt the frustration communicated by participants from the unreformed sections. We speculate that the ability to progress through the assignment, and having smaller but more frequent assignments, mitigated their frustration.

The *Practice* and *Feedback* themes broadly manifest as inextricably linked, beyond the context of reading assignments. When both are present in some form, they appear to advance learning and prompt positive affective outcomes. However, if one or both are lacking, it negatively impacts learning and affective outcomes:

“[···] she kind of got on our level [···] whereas, not a lot of the teachers are like that in a sense where they’re kind of more just like, ‘Well, if you’re not figuring it out, try harder.’ It’s like, ‘Well, can’t try harder if I don’t know what the heck it is that I don’t know’.” (R‑2)

“it’s like they expect because you’re in these classes that you’re already supposed to know what’s going on, and we don’t. [···] Like, that’s why I’m taking the class [···] there was no way for you to break into there, I don’t know—baby steps, nothing—it was just automatically difficult.” (UB)

Consequently, in Figure 2, we indicate *Practice* and *Feedback* together as prompting positively-oriented affective outcomes when present, or prompting negative-oriented affective outcomes when one or both is absent. We use the phrase “practice-feedback opportunity” to describe instances where students have the opportunity to engage in practice and receive feedback they are able to use to advance their learning. Importantly, the existence of a potential source of feedback does not necessarily mean all students are able to use that feedback to advance their learning. Consequently, a given practice-feedback opportunity may be accessible for some students but inaccessible for others.

### Self-Regulation Strategies

The *Self-Regulation Strategies* theme reflects participants’ descriptions of ways they support their own learning independently, outside of course activities like homework and lecture/office hours attendance. Participants who found strategies they could use independently to support their learning generally described this in a positive light. In some cases, this manifested as summarizing and solidifying one’s understanding through explaining it to others:

“I would try to explain what I learned to my family but in Spanish, because they don’t speak English. And that added a new depth to my understanding because I would switch vocabularies from English to Spanish and familiarize myself more with the material.” (R‑1).

In other cases, participants recounted instances where they were able to independently create their own practice-feedback opportunities:

“[in the homework platform] I would do the like ‘show me a different question but the same format with different numbers,’ so I would end up doing it over again until I could actually do it on my own.” (UA‑2)

“I just do more myself in reviewing [···] like okay this was an example he gave us so I’ve got written down [···] I’ve got the answer I know is correct and then just trying to work back” (UB)

However, other participants described trying to independently support their own learning, but finding their efforts ineffective:

“I did not know what to take notes on. So I was discouraged because I’m sitting here for one chapter and I’ve been taking notes for five hours. And I didn’t know what to write down.” (UA‑2)

“I tried notecards but it was really just like rewriting the exact same thing in the lecture so that was too much.” (UB).

In this way, a participant’s ability to use effective self-regulation strategies contributed to more positive affective outcomes, promoting confidence in one’s knowledge and/or ability to succeed. In contrast, when participants struggled to identify self-regulation strategies that were effective, that contributed to more negative affective outcomes such as feeling overwhelmed or unable to learn. Thus, in Figure 2 we also represent *Self-Regulation Strategies* as promoting positively oriented or negatively oriented affective outcomes when present or absent, respectively.

We notice the relative importance of participants’ (non)­use of self-regulation strategies appears to vary across sections. In the unreformed sections, participants comment both on the utility of effective independent self-regulation strategies, and of difficulties stemming from a lack of such practices. However, in the reformed section, participants occasionally mention the utility of effective independent self-regulation strategies, but there is no notable discussion of difficulties arising from the absence of such practices. In this way, the reformed course structure appears to reduce how strongly the lack of independent *Self-Regulation Strategies* influences participants’ affective outcomes.

### Student-Centered Strategies

The *Student-Centered Strategies* theme reflects participants’ descriptions of instructional practices that had a positive impact on their learning. We include practices used by supplemental instructors, who were undergraduates who had previously taken the course and were employed to lead optional interactive study sessions outside of class meeting times. Supplemental instruction was heavily advertised to students in the unreformed course sessions, but not to students in the reformed section because that course piloted a new structure unfamiliar to the supplemental instructors.

This theme manifests in a variety of ways. It includes instances where participants describe content being introduced or explained in a way that is responsive to and emphasizes their understanding:

“I knew that [instructor] took into consideration that the way that the text words specific things can be very [···] confusing. Because there’s a lot of big words that we might not understand and it can get really annoying. So, her putting the videos in place to that was really helpful and being able to actually like, intake the content and understand it because it was in a way that, one, was visual, so we were able to see it, but it also kind of made sense to us because it was explained in simple words rather than big words. And if there were big words, they were explained” (R‑1)

“[Instructor’s] lectures were really easy to listen to and focus [···]­everything she did was very clear and concise. And if it was clear that people weren’t understanding, she would adapt and change how she was teaching.” (R‑2)

“[supplemental instructor] was able [···] to put it into kind of, I don’t know how to--not chemistry language, [but] English. It made sense” (UA‑1)

“[with the supplemental instructor], you were able to retain the information more [···] you were learning from a peer, because she was like one of us basically. So it was [···] real life experiences, like being able to explain stuff how [supplemental instructor] knows it, but how we will also understand it” (UB)

Sometimes, this theme manifests through opportunities to actively practice and discuss the material with others:

“[instructor] was also really good at writing on the board and not just only showing slides, like giving examples and having us work through and being like, ‘Okay, talk to your neighbor. Figure this out.’ Rather than just being like, ‘Okay, this is a slide. Okay, here’s the answer.’ It’s not really helpful if it doesn’t really push you to do stuff” (R‑2)

“[supplemental instructor] would put different questions up on the board. [···] she would split us up, so we had our own little groups [···] I really liked it because we were able to talk about it, and we were able to help each other out.” (UA‑1)

or through pedagogical strategies that structurally space students’ practice:

“One of the things that she did that was very helpful personally for me was she kind of spread out the homework on an everyday basis rather than the end of the week.” (R-1)

Interestingly, while only the reformed section structurally spaced out students’ practice, participants from unreformed sections independently expressed a desire for this:

“we had one assignment due per week [···] if she [had] split it up and given it due like one day 25 questions and then the next day 25, that would have helped me” (UA‑1)

At other times, *Student-Centered Strategies* manifest as opportunities for students to develop an interest in the material, often through connections to prior knowledge or real-life examples:

“[instructor]’d raise some sort of question and maybe we would have to guess what the outcome of doing something would be and that would relate to the big concept of the day. And sometimes it’s not very apparent what’s going to happen so you kind of have to [···] think back, use your knowledge to put together an educated guess and a lot of the time the educated guess is not right at all. [···] I think that it sticks with you more than [···] if she hadn’t kind of challenged your preconceived notions.” (R‑1)

“She would use examples from everyday life to try and explain stuff, which made it a lot easier.” (R‑2)

“[···] with certain topics [supplemental instructor] was able to like relate it to like, the world itself so it would be easier to grasp that information” (UB)

Another way this theme manifests is through the instructor cultivating a culture of communication to be responsive to students’ needs; this is only mentioned by participants from the reformed section. This includes explaining the purpose of course practices:

“[···] one really great initiative was she would even just vocally say during class that a lot of her teaching styles and things that she did was based on research of what is found to work the best, and there were a lot of different things she did to try and make sure she was, I guess, hitting as many people as possible.” (R‑1)

and soliciting student feedback on various aspects of the course. One participant highlights an example in response to students’ having difficulty reading the whiteboard:

“she’d also asked for feedback all the time, which was really nice. With the board issue because of the marker she asked like, ‘What do you guys want us to use? Like me writing on a piece of paper and have it projected onto the wall?’ So, she always, always asked for feedback, which was nice. Again, it just made chemistry easier, more enjoyable” (R‑2)

While the deliberate practice framework focused our attention on exploring student perceptions of how structural attributes of the course promoted motivation and learning, we noticed a social component that appears to play a role in how participants describe their engagement with student-centered practices. Participants from the reformed course remark on how their outlook and engagement was impacted by their perception of how the instructor cared about their learning:

“This class definitely changed my opinion of chemistry, because, well, it was a good teacher. Because [instructor], you could tell she cared about us, tried to make sure that we understood it, everyone in the classroom, despite how many students she had. I saw that effort there and now I really do enjoy it.” (R‑1)

“[···] it was getting towards the end of the semester. So a lot of us were kind of starting to get into the mindset of checking out, getting ready for winter break. But I feel like just because she had kind of already gained that respect and built up that respect, even if the material was hard, we all just kind of made sure we kept our heads in the game for the sake of not only us but also for the respect for her. Because she had always been in the game.” (R‑1)

“Having a teacher that cares is probably the most important thing because it makes you want to engage.” (R‑2)

To be clear: we assume all instructors care about students’ learning and want students to learn. However, some participants describe experiences with instructors in other courses that indicate how instructors’ care is not necessarily obvious to students:

“it’s just kind of a nightmare when your [···] science classes are taught by somebody who is just kind of not great in terms of teaching or attitude” (R‑1)

“[this term] my professors are only there because they want to keep their research going and you can totally tell that with their passion towards the class.” (R‑2)

Overall, the *Student-Centered Strategies* theme often arises in discussion among participants from the reformed course section. When mentioned by participants in the unreformed sections, it is nearly exclusively about the optional supplemental instruction sessions. Overwhelmingly, participants mention this theme in a positive light. In a few isolated cases, participants comment on how experiences might have been improved through the incorporation of *Student-Centered Strategies*, but we do not recognize instances where participants draw a clear causal tie between the absence of these strategies and their negative affective outcomes. Thus, in Figure 2 we represent *Student-Centered Strategies* only as promoting positively oriented affective outcomes when present, and not as prompting negatively oriented affective outcomes when absent.

### Structural, Logistical Barriers

The *Structural, Logistical* *Barriers* theme reflects participants’ descriptions of challenges that stem from institutional structures or systems. These include several factors over which the instructor has limited control, such as the large class size, homework platform, or the extensive amount of content the course is expected to cover:

“[···] sometimes it was kind of impossible to figure out what was the format that the homework would accept. Even if you had the right answer, just figuring out how the formatting worked so that it wouldn’t mark you wrong” (R‑1)

“I feel like there was sometimes when she should’ve spent more time on certain subjects.” (R‑2)

“I was just terrified because I had never been inside a huge lecture hall. [···] It was huge. [···] and I was like, “I’m going to have to know everything.” (UA‑1)

“It was kind of overwhelming. It was like it was just so many people and then it’d be so big and so much content to go through.” (UA‑2)

“[···] bigger classrooms are more of a distraction [···] I put more responsibility on myself too when it’s smaller” (UB)

These factors also had an influence on other course aspects, such as the amount of homework and sense of “connectedness” between course topics or assignments:

“it kind of seemed like one person was in charge of the readings, one was in charge of the homework, one made the slides, nothing full-circle connected them” (UA‑1)

“when I get the test, it’s just a lot of those questions have nothing to do with what [we] learned.” (UA‑2)

“some of [the homework assignments] were like the 70, 80 questions each, so I was at it for like hours, so I kind of just stopped doing it.” (UB)

In this same vein, participants describe logistical barriers they encountered due to their personal circumstances:

“the way my schedule’s set up, I couldn’t visit her office hours on Wednesdays because [··· of] my math class” (R‑1)

“the review session [···] never worked with my schedule because I always had class during those times.” (UA‑1)

“[···] it was just personally hard for me to really comprehend what was being said. And my [supplemental instructor], her times didn’t always match up with my times, so I couldn’t go [···] so I kind felt on my own.” (UB)

In this way, scheduling conflicts and other courses can prevent students from engaging in practices that could help them compensate or overcome these challenges.

While participants exclusively linked this theme to negative affective outcomes, there is still some complexity in how it frames students’ perceptions. For example, participants from all sections unanimously describe the large lecture format as a barrier to their learning that prompts negative affective outcomes. However, participants describe their perception of the instructor’s practices within the context of the course size, which influences their perception. For example:

“it always sticks out to me, it was fourth week, before midterms, and we were getting our first test back. I walked down to her and I had never spoken to her since it was such a big class and she knew my name, and it was really--it was just meaningful to me that she already knew my name.” (R‑2)

In this case, the large class size means that this participant did not receive any individualized feedback or interact directly with the instructor. However, this does not appear to preclude the participant from feeling a sense of connection with the instructor: the instructor’s attempt to learn their names seems to make more of an impact *because of* the large class size. This suggests that instructors do maintain some influence over how structural barriers impact students’ overall affective outcomes, even when these structural barriers are outside of instructors’ control.

## Discussion

Here we discuss how the thematic map presented in Figure 2 can be used to understand how the course design influences students’ attitudes (Research Question 1). We also discuss how this aligns with tenants of the deliberate practice framework used to structure the reformed course (Research Question 2). While much of this discussion focuses on “course-level” differences, we also unpack how the thematic map can be used to explain why two individuals in the same course section may experience the course differently, resulting in different affective outcomes. We then discuss implications of these findings and their limitations. To provide the reader as much insight as possible into the data informing these interpretations, where relevant we refer back to the results section and/or incorporate additional illustrative quotes.

### Alignment with Deliberate Practice Framework

As we have described, author N.M.J. designed the reformed course structure based on deliberate practice tenants. This included efforts to promote motivation through pedagogical transparency, explicitly connecting the content to students’ real-world experiences and goals, and frequent practice-feedback opportunities in the form of pre-class assignments, in-class active practice, and homework assignments, and spacing out practice-feedback opportunities. Participants from the reformed course commonly highlight these same aspects of the course structure and comment on how they promoted their motivation and ability to learn. This aligns with the findings of our prior study,^20^ which found an association between the reformed course section and student learning outcomes. The qualitative results presented here suggest a causal tie between these course features and their learning, in alignment with the reform’s intentions. Interestingly, we can also triangulate the role that sources of motivation, practice, and feedback played through the perceptions of participants from the unreformed course sections. In the unreformed sections, it is less common for participants to describe experiences that developed their motivation or provided practice-feedback opportunities, but those that do generally describe those experiences to positively impact their affective outcomes.

We view student perceptions, feelings, and attitudes to be directly connected to content learning, which can be seen through the deliberate practice framework’s focus on student motivation. In alignment with this, participants across all sections describe a close connection between their course-based learning experiences and their broader attitudes toward and self-efficacy in chemistry as a discipline. Participants who describe more positive course experiences also tend to articulate a more neutral-to-positive orientation in their attitudes toward chemistry. Similarly, participants who describe more negative course experiences tend to articulate more negatively oriented chemistry attitudes. Importantly, this does not appear to correlate to perceptions that a course is “easier” or “harder.” Rather, participant attitudes appear more associated with perceptions of their time and/or effort being worthwhile. For example, all participants from the unreformed sections describe the reading assignments as highly time intensive, but those who felt their time and effort ‘paid off’ by reinforcing or extending their learning generally describe the assignment in neutral terms, e.g: *“I was kind of neutral on it”* (UB). In contrast, students who did not feel the time and effort benefitted their learning describe the assignment in highly negative terms, e.g.: *“it was like just killing me”* (UB). In this way, the perception of difficulty appears to matter less than the perception of it being possible to succeed.

In this same vein, the fact that participants describe instances where they disengaged (e.g., not completing reading assignments), does not mean a lack of motivation to learn or unwillingness to put in effort. As an illustrative example: focus group UB engaged in an extended discussion about frustrations about how the course lecture nearly exclusively involved reading off slides: *“I could do that at home, like, why am I paying you to read to me? I know how to read.”* A focal point in this frustration was that time spent in class was not perceived to provide added value for their learning: *“When they post those PowerPoints I’m like—well okay, why am I going to class?”; “[···] it kind of felt like, well, go or don’t go, it doesn’t matter. I’m getting the same amount of education.”* There was widespread agreement of this, and then without prompting participants began to discuss possible solutions, all of which evoke practices mentioned in the *Student-Centered Practices* theme that involve more effort from students: *“in the beginning of class maybe like a five question quiz [···] of something you learned in the class before.”* ; *“Maybe they could, like, get rid of having to buy the textbook [···] and use that money toward like the clicker program [···] so that could boost participation”*.

Thus, we interpret the *“Don’t learn it. Do what you need to do to pass”* mindset described by participants to be a result of the circumstances they experienced, not their preferred outcome. In other words: when it did not feel feasible to learn, participants stopped focusing on learning and instead focused on getting through or ‘surviving’ the course. We also capture indications of the converse: participants in the reformed course described feeling able to learn and consequently expressed a desire and sense of responsibility to put in effort:

“Chemistry was my only class on Friday so sometimes I didn’t feel like going but I would still come. [···] Because I didn’t want to be behind on any material” (R‑1)

“[···] she did everything above and beyond on her part as a teacher, so it was just on us, and the part of the students, to do what we have to do” (R-1)

“[···] out of all of [my classes], her class was the one I hated missing. Just because I learned the most” (R‑2)

This is consistent with our theoretical framing of student attitudes as a broad umbrella within which student affect can influence learning via motivation to engage in behaviors that involve effortful practice.

In addition to the structural features of the course that participants describe to influence their motivation, we also note that participants emphasized the role played by their perception of how much the instructors cared. When participants perceived their instructor to care about them and their learning, they were more motivated to engage with the course. When participants did not perceive their instructor to care, they were less motivated. This suggests the importance of instructors making their care visible to students. We do not believe this necessarily involves dramatic changes; participants demonstrated awareness of the varied demands on instructors, and did not appear have unreasonable expectations:

“I definitely appreciate--because I know professors, they’re doing research and they’re doing all this stuff that’s taking up all their time and it’s like ten different jobs in one.” (R‑1)

“It needs to be, like, you don’t hate coming to class. It’s like, you’re a teacher. It’s your job, you signed up for this. You should not be hating your life as much as you appear to be” (R‑2)

This suggests that relatively simple actions could make instructors’ care more transparent to students and thereby reinforce students’ existing motivation to engage in learning.

### Variation among Affective Outcome Paths

Participants’ experiences are complex and multifaceted. All participants describe some course attributes that create challenges for their learning, and most participants describe some factors that positively affected them. However, there are notable differences in how commonly participants report positive or negative experiences. Participants from the reformed course less often discussed negative experiences and more often discussed a variety of positive experiences, which were met with greater agreement from other focus group participants. The unreformed course focus groups displayed the reverse trend, with more prevalent negative experiences and less prevalent positive experiences that, when they existed, were primarily associated with supplemental instruction.

The thematic map presented in Figure 2 is a useful tool for explaining this variation, as it represents the “landscape” of possible paths an individual’s experiences may take, depending on what factors are present and accessible to that individual. Figure 3 provides illustrative examples of four different hypothetical “paths” an individual’s experience could take on this landscape.

**Figure 3:**
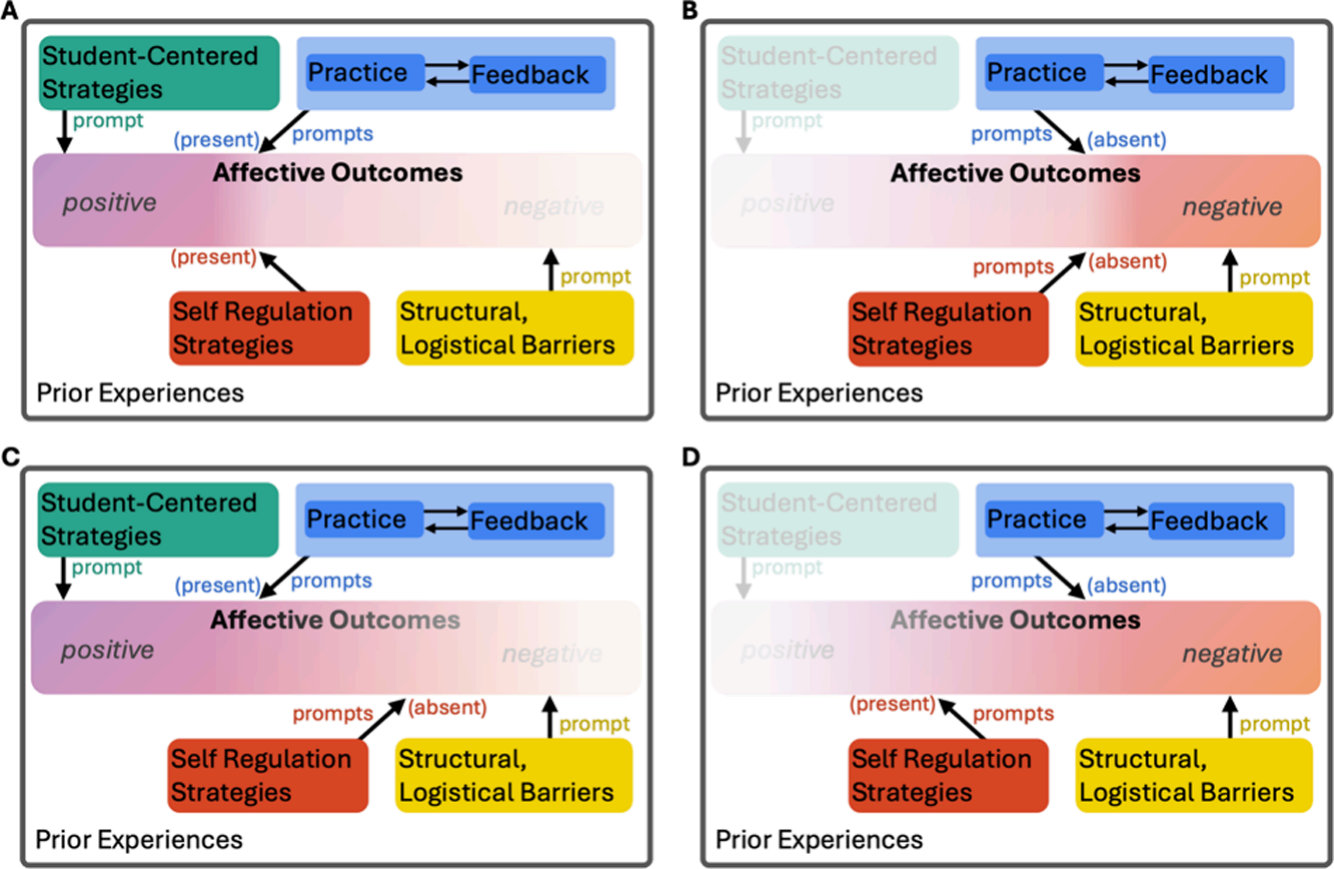
Example paths a student could experience within the thematic map landscape. Affective outcomes are a spectrum; we use a transparency gradient to convey general tendencies toward more positively oriented or more negatively oriented affective outcomes. Higher color intensity (lower transparency) qualitatively indicates a stronger relative tendency.

Figure 3A and Figure 3C represent cases where a student takes a course that incorporates student-centered strategies and practice-feedback opportunities. These attributes prompt positively oriented affective outcomes despite the presence of structural or logistical barriers faced by the student. In Figure 3A, the student also has an ability to independently employ self-regulation strategies, which further accentuates the positive orientation of their affective outcomes. In Figure 3C, the student is not able to independently employ self-regulation strategies, but the influence of the student-centered strategies and practice-feedback opportunities still result in a relatively neutral-to-positive affective outcome.

In contrast, Figure 3B,D depicts scenarios where a student takes a course that does not incorporate student-centered strategies, and practice-feedback opportunities are either not present or the feedback is provided in such a way that the student does not find it accessible. In these cases, the absence of practice-feedback opportunities and the presence of structural, logistical barriers prompt more negatively oriented affective outcomes. In Figure 3B, the student is not able to leverage self-regulation strategies to compensate, which further accentuates the negative orientation of their outcomes. In Figure 3D, the student can leverage self-regulation strategies, which mitigates the impact of other factors and shifts affective outcomes accordingly.

In our results, we observe cases similar to each of these hypothetical paths. For example, some participants from the unreformed sections describe ways they independently created practice-feedback opportunities (a manifestation of *Self-Regulation Strategies*) or had schedules that allowed them to attend supplemental instruction (an example of how their *Structural, Logistical Barriers* did not prevent access to effective *Practic*e-*Feedback* opportunities). These participants generally report having maintained or slightly improved their chemistry attitudes and confidence (*Affective Outcomes*) over the span of the course.

Participants from the reformed section also describe impacts of schedule conflicts and course attributes such as class size (manifestations of *Structural, Logistical Barriers*), or instances where they did not perceive an assignment to impact their learning (an ineffective *Practice*-*Feedback* opportunity). However, these participants also describe other attributes of the course that helped them compensate for these challenges, such working practice problems in class (an effective *Practice-Feedback* opportunity) or how the instructor responded to their questions and/or points of confusion (manifestations of *Student-Centered Strategies)*.

In this way, it appears that by using *Student-Centered Strategies* and incorporating a variety of in- and out-of-class *Practice-Feedback* opportunities, the reformed course structure provided more possible paths leading to positively oriented affective outcomes. This increases the likelihood that a student will find at least one of these paths accessible, essentially providing more “insulation” from the negative repercussions that could result from the *Structural, Logistical Barriers* they experience, how their *Prior Experiences* may set them up for the course, and any difficulties in independently finding effective *Self-Regulation Strategies*. In comparison, the unreformed courses appear to offer fewer paths leading to positively-oriented affective outcomes, making the accessibility or inaccessibility of any one path more influential, and thus making students more sensitively impacted by differences in their *Structural, Logistical Barriers*, *Prior Experiences,* and use of effective *Self-Regulation Strategies*. In this way, the reformed course section appears to more consistently influence students’ affective outcomes in a positive direction, compared to the unreformed course sections.

### Equity Implications

Importantly, course structures that rely more sensitively on students’ life circumstances (via the *Structural, Logistical Barriers* they navigate, their *Prior Experiences*, and independent use of effective *Self-Regulation Strategies*) can be expected to perpetuate existing societal inequities. For example, students whose *Prior Experiences* are such that they must co-enroll in other preparatory or remedial college courses face more *Structural, Logistical Barriers* as a result—particularly time pressure:

“I think outside courses also caused difficulty for me because I had six classes that semester, and one of them was the basic math where they require you to be in their math lab for three hours and that kind of stuff.” (R‑2)

“if you’re taking [this course], most of the time you’re taking [preparatory math course]. [That math course] already has like 100 questions itself so its like, you gotta get your math hours, and I’m just like—who has time to be doing all of this chemistry [···] and then go do all of this math. Like we going to have to split it between the two” (UB)

In this way, we speculate that it is reasonable to expect that the students who would likely most benefit from *Student-Centered Strategies* and *Practice-Feedback* opportunities are disproportionately among the students least likely to be able to attend out-of-class activities such as office hours and supplemental instruction. This is consistent with contradictory findings in the literature about the effectiveness of remedial coursework^64–66^ and the possibility of remedial coursework hindering students’ educational outcomes.^67,68^ This increases the importance of in-class practice-feedback opportunities and student-centered teaching strategies.

In sum: we argue that courses can better support positive affective outcomes among all students by reducing how sensitive students’ course experiences are to their life circumstances. Here, that was accomplished by the reformed course structure incorporating deliberate practice tenants, resulting in multiple forms of *Student-Centered Strategies* and *Practice-Feedback* opportunities integrated in-class and out-of-class.

### Limits and Scope

In this study, we investigated students’ perceptions of how course structures impacted their experiences and attitudes toward chemistry. It is possible that participants’ perceptions were not complete and/or may vary over time. For example, participant descriptions of how prior experiences impacted their learning focused exclusively on high school math and science preparation. However, there is evidence that other aspects of prior life experiences also play a role in and can be an asset for student learning.^69^ The fact that participants did not mention these aspects does not mean they do not play a role; it could be that participants are not metacognitively aware of these factors, that they simply did not occur to the participants at the time, and/or that participants found them too personal to share in a focus group format. We cannot rule out any of these factors, but we suspect the latter option is less likely because in all focus groups multiple students mentioned (and appeared comfortable discussing) instances of struggle, failure, and/or illness.

Additionally, affective outcomes appeared to be closely related to participants’ perceptions of their learning, but student perceptions are not necessarily accurate measures of their learning.^70^ However, informed by social constructivist and deliberate theories of learning, we view student affect to play an important role in how students engage in learning. Thus, we intend for this work to provide complementary insight to our prior publication that focuses more on cognitive outcomes.

## Conclusions

In this study, we sought to understand the relationships between introductory chemistry course structure and students’ attitudes toward chemistry. In particular, we were interested in understanding causal mechanisms that may have contributed to how students enrolled in a reformed introductory chemistry section had improved attitudes toward chemistry compared to students in concurrent, unreformed sections.

Through our analysis of focus group discussions, we developed a thematic map illustrating mechanisms that participants described that connect personal factors and course structure to their chemistry attitudes. All participants described structural factors that impaired their ability to learn and negatively impacted their course experiences, such as class size and time constraints. However, participants also comment positively on experiences that provided the opportunity to engage in practice and receive feedback, as well as instruction that incorporated student-centered practices such as building upon students’ prior knowledge and using real-world examples. Participants described these experiences in positive terms, remarking on how it made learning appear more feasible and cultivated their interest and motivation. Participants reported that their course experiences directly impacted their broader attitudes toward chemistry as a discipline.

While positive experiences are described in all focus groups, they manifest differently for the reformed and unreformed course sections. Reformed course participants more positively discussed practice-feedback opportunities and student-centered teaching strategies, which manifested in a wider variety of ways in and out of class. Unreformed course participants less often discussed instances of practice-feedback opportunities and student-centered teaching strategies, and those that were discussed mainly occurred outside of class and/or involved participants creating those opportunities independently through self-regulation strategies. Additionally, negative views—and a wider variety of views—were more prevalent among the unreformed course sections.

Participants’ descriptions of practice-feedback opportunities and motivation as a result of student-centered teaching strategies align well with the deliberate practice framework that informed the course reform. We argue that, by using this framework to intentionally design these practices into the course structure in multiple ways, the reformed course increased the likelihood that students were able to engage with and benefit from these practices. In contrast, we argue the unreformed course participants’ experiences varied more and were more often negative because their ability to engage with these practices relied more heavily on their prior experiences and the structural and logistical barriers they navigated.

This work contributes insights into how a deliberate practice-informed course reform improved students’ attitudes toward chemistry, with implications for improving introductory chemistry students’ experiences and outcomes. These findings also illustrate how one’s thoughts and feelings directly influence their learning, underscoring the importance of considering students’ perceptions and affective responses in education research. Furthermore, we find participants’ attitudes toward chemistry *as a discipline* were heavily influenced by their course experience. This suggests that even though prior experiences play a role in setting participants attitudes, introductory chemistry courses present a unique “high risk/high reward” opportunity: positive introductory chemistry course experiences can catalyzed students’ confidence, interest, and desire to learn in chemistry; however, negative introductory chemistry course experiences can impair students’ confidence and interest to the point of causing students to alter their career goals entirely to avoid future chemistry courses.

In this way, these findings highlight the importance of mitigating the extent to which a student’s course experience relies on life circumstances such as the ability to attend out-of-class meetings or independently create practice-feedback opportunities. Instead, these findings suggest that structurally incorporating student-centered teaching strategies and practice-feedback opportunities into the course can more equitably improve students’ chemistry attitudes.

## Supplementary Material



## Data Availability

Deidentified data sets used in the current study are available from the corresponding author on reasonable request.

## Notes

Codebook including all open codes used to inform the development of themes and additional narrative descriptions of how these themes were observed to manifest throughout the focus groups (PDF)

## Abbreviations

FG: focus group
LMS: learning management system
R-1: first focus group of participants from Section R
R-2: second focus group of participants from Section R
UA-1: first focus group of participants from Section UA
UA-2: second focus group of participants from Section UA
UB-1: first (only) focus group of participants from Section UB.
