# Supplementary material for: This Class Definitely Changed My Opinion of Chemistry: How a Pedagogical Course Reform Improved Students’ Chemistry Attitudes
Source: ACS Omega. 2025 Jul 30;10(31):34506–21. doi: 10.1021/acsomega.5c02931 (PMC12355253; doi:10.1021/acsomega.5c02931)
Supplement: Supplementary file 1 [file ao5c02931_si_001.pdf]

Supplemental Information for: ‘This class definitely changed my opinion of chemistry’: How a pedagogical course reform improved students’ chemistry attitudes

Below we provide the open codes developed in our codebook, and how they informed the development of each theme. To be as transparent as possible, we also provide a description of how we observed these codes manifested throughout the focus groups.

Affective Outcomes

Table S1: Constituent codes of the descriptive theme: *Affective Outcomes*

Code	Definition	Illustrative Quotes
“confident”	belief in one’s subject knowledge and ability to learn	“Coming out [of the class], I did feel confident in my chemistry abilities, to do well in chemistry” (R-1)<br><br>“now I feel like […] I can handle any chemistry class." (R-1)<br><br>“I learned that I was capable of learning in the science[s].” (R-2)
desire to learn	interest in learning course content and/or more about the subject	“I started to look forward to [the next chemistry course], and beyond” (R-1)<br><br>“I like the classes where the teacher makes me want to come to class […] I loved chemistry because I wanted to come and I wanted to learn more” (R-2)<br><br>“before I didn’t really like chem, but now like I love it, […] I wish I could take more classes with it” (UB)
“do what you need to”	focus on fulfilling the minimum requirements to move forward	“there's a lot of just, ‘get through it. Don't learn it. Do what you need to do to pass’ kind of thing.” (UA-1)<br><br>“I was just like, I'll take a low C. I just need to pass” (UA-1)
“forced”	describing a need to do things despite a lack of desire or interest	“we would have to force ourselves to listen, take notes, ask questions” (UA-2)<br><br>“if you get points for like coming to class […] that’ll force people to come” (UB)
isolated	Feeling unconnected with others in the course	“there isn't really any interaction with anyone during the lecture” (UA-1)<br><br>“I didn't really have any one-on-one relationships in association with this class.” (UA-2)

		“there needs to be more like interaction […] [students] need that kind of connection with others so that they can get help” (UB)
“manageable”	perception that course tasks and assignments are feasible	“It was manageable as long as you put the effort in” (R-1)<br><br>“Homework every other day that we can come in and for help and it’s more manageable” (R-1)
“overwhelming”	negative feelings about the large scale of the course, content, or interpersonal environment	“I was just terrified because I had never been inside a huge lecture hall. […] It was huge. [...] and I was like, "I'm going to have to know everything." (UA-1)<br><br>“there was just so much material to cover and everything. We would look at the PowerPoints, and there would be 100 plus slides. […] it was just overwhelming” (UA-1)<br><br>“it was kind of overwhelming. It was like it was just so many people and then it'd be so big and so much content to go through.” (UA-2)

In all focus groups, there was some discussion of affective outcomes that were generally positive (e.g., “confident”, desire to learn). However, the focus group discussions with students from the reformed section (R-1, R-2) more frequently recounted positive affective outcomes, and more frequently expressed a consensus toward those outcomes, with many or all focus group participants expressing agreement with one participant would remark on a positive affective outcome. In the focus group discussions among students in the unreformed sections (UA-1, UA-2, UB), reports of positive affective outcomes were more rare and other participants more commonly expressed opposing viewpoints.

Similarly, in all focus groups there was mention of affection outcomes that were more negatively oriented (e.g., “overwhelming”). However, such mentions were rare in focus groups from the reformed course (R-1, R-2) and generally arose during discussions of structural course elements such as the homework platform and/or time limitations due to other courses; this is discussed in more detail in the theme *Logistical Structural Barriers*. In focus group discussions with students from the unreformed sections (UA-1, UA-2, UB), negative affective outcomes were commonly shared an generally met with widespread agreement.

Feedback

Table S2: Constituent codes of the descriptive theme: *Feedback*

Code	Definition	Illustrative Quotes
asking questions in class	Ability or inability to get responses to course- or subject-related questions during course sessions	"Even in a class that big she was always willing to answer questions. […] When we did individual work, she always came up to us if we had a question and I really appreciate that." (R-2)<br><br>"no one really asked questions because she was just zooming through […] next thing you know, she's on a totally different topic so it doesn't even make sense to ask anymore." (UA-1)<br><br>"[…] in lectures, it's kind of hard to ask questions. And then 50 minutes, you don't have enough time to really ask questions" (UA-2)<br><br>"when you're in a big lecture hall—it makes you afraid to like, ask questions" (UB)
feedback	constructive comments or information (or the lack thereof) about one's understanding or work	"sometimes she would even make you explain why you think you're right or wrong just to make sure you understand the concept. […] if you got it wrong she'll explain why this is right and why this is wrong, this is a common mistake, how to avoid this, and stuff like that." (R-1)<br><br>"she'd always know what people are usually having trouble with based on test results or reading responses. She'd know what to help people on" (R-1)<br><br>"on the homework […] you could check your answer to make sure it's good, so if it is, then you can move on, and if not, then you're like, 'Okay, I need to revisit my notes'." (R-2)<br><br>"I'm like, 'I don't know I did wrong. How am I supposed to study off of this to figure out what to do next?'" (UA-1)
out of class help	space for students to seek instructional help outside of course sessions	"I got everything mixed up, and I went to her office hours multiple times in a week[…] she was really helpful." (R-1)<br><br>"if I was struggling with a homework question, I knew I could go to her before class and […] after class." (R-2)<br><br>"the only real way I learned […] was the few times I could go to the [supplemental instruction] leaders like after hours" (UB)

We noted a similar trend as observed in the *Affective Outcomes* theme: participants in R-1 and R-2 discussed receiving a wider variety of feedback on their learning, and participants in UA-1, UA-2, and UB more often recounted challenges with obtaining feedback on their learning.

Logistical Structural Barriers

Table S3: Constituent codes of the descriptive theme: *Logistical Structural Barriers*

Code	Definition	Illustrative Quotes
a lot of homework	Difficulty due to the scope of course work that must be completed out of class	"we had one assignment due per week and so many questions" (UA-1)<br><br>"some of them were like the 70, 80 questions each, so I was at it for like hours, so I kind of just stopped doing it." (UB)
class size	challenges caused by the number of students in the class	"[you] don't interact in class, it's too big" (UA-1)<br><br>"The class size was [..] kind of overwhelming. It was like it was just so many people" (UA-2)<br><br>"bigger classrooms are more of a distraction […] I put more responsibility on myself too when it's smaller" (UB)
grading or homework system challenges	Challenges encountered as a result of grading mistakes and/or difficulties with the homework system	"sometimes it was kind of impossible to figure out what was the format that the homework would accept. Even if you had the right answer, just figuring out how the formatting worked so that it wouldn't mark you wrong" (R-1)<br><br>"the whole [homework] program was kind of wonky at times" (UA-1)<br><br>"he put it in like I had got a 78 or 79 but in actuality I had like an 85 and [when I asked] s/he was like, ah yea" (UB)
need more time	Sense that there is insufficient time to cover necessary course content	"more time [in class] […] maybe like an hour and 15 [minute] class, that maybe will help." (R-1)<br><br>"I feel like there was sometimes when she should've spent more time on certain subjects." (R-2)<br><br>"she did go really, really fast, […] but sometimes her going so fast I would miss kind of certain things" (UA-2)
pre-req	Course is required to continue in the desired major or program	"I needed [it] for my major" (R-2)<br><br>"I'm pre-med, that was kind of the prereq for all the other science classes" (UA-2)<br><br>"you can't even go to like a different course for your major—it's like you gotta take this [course] and you pass it, or you don't take anything else." (UB)
scheduling challenges	Barriers in participation that arose from challenges or conflicts in one's schedule	"the way my schedule's set up, I couldn't visit her office hours on Wednesdays because [… of] my math class" (R-1)<br><br>"I think outside courses also caused difficulty for me because I had six classes that semester, and one of them […] require[d] you to be in their math lab for three hours" (R-2)<br><br>"the review session […] never worked with my schedule because I always had class during those times." (UA-1)<br><br>"I only used [supplemental instruction] like once […] with scheduling, couldn't get to her. But she was helpful" (UB)

unconnected	Lack of clear relationships between course topics or assignments	“it kind of seemed like one person was in charge of the readings, one was in charge of the homework, one made the slides-- nothing full-circle connected them” (UA-1)<br><br>“when I get the test is just a lot of those questions have nothing to do with what [we] learned.” (UA-2)

Codes in this theme arose relatively consistently across all focus groups, regardless of course section. However, we noticed differences in how they manifested as a function of course section. For focus groups R-1 and R-2, these discussion points were more or less only present when responding to prompts about how they might change the course to improve their experiences and/or when something felt challenging. For the other focus groups (UA-1, UA-2, UB), codes in this theme arose frequently throughout the discussion in response to a wider variety of prompts—even in response to prompts such as “Tell me about a time when you felt you were able to learn”.

Self-Regulation Strategies

Table S4: Constituent codes of the descriptive theme: *Self-regulation strategies*.

Code	Definition	Illustrative Quotes
distilling information	Summarizing or explaining concepts or ideas to solidify one’s understanding	“I would try to explain what I learned to my family but in Spanish, because they don't speak English. And that added a new depth to my understanding because I would switch vocabularies from English to Spanish and familiarize myself more with the material.” (R-1)<br><br>“Once I had to explain and did it a few times I would be like, ‘now I get it’.” (R-2)<br><br>“Even though the [reading assignments] were long I actually used [them] to take notes” (UA-2)
creating ways to practice	students finding their own avenues for additional practice of course material	“I just did the assistant help, or whatever, on the homework. […] I'd retry the question off of that, just match them up basically. […] You can just change the numbers. So that helped a lot” (UA-1)<br><br>“I would do the like ‘show me a different question but the same format with different

		numbers,' so I would end up doing it over again until I could actually do it on my own." (UA-2)<br><br>"I just do more myself in reviewing […] like okay this was an example he gave us so I've got written down […] I've got the answer I know is correct and then just trying to work back" (UB)
spacing work out	students breaking the work into smaller pieces spread over time	"[I] try and study at least for 20 minutes every day, reviewing to make sure I knew the material rather than waiting until the last minute" (R-1)<br><br>"she'll be on one section in the lecture […] [and I'll] do that part of the [homework] the same day. […] so I'm not procrastinating […] versus waiting until mine gets due, and then I forget everything." (UA-2)

We noted that it was more common for participants from the unreformed course sections (focus groups UA-1, UA-2, UB) to describe use of self-regulation strategies, and participants from these sections described using a wider variety of such strategies. For participants from the reformed course section (R-1, R-2), they more commonly described engaging in practices in class or with the instructor, such as working a practice problem with a partner in class or in office hours. Such instances were not considered "self-regulation strategies," since they were not performed independently of course activities. There, however, were a few instances where focus group participants from the reformed section recounted strategies performed independently—these instances described strategies similar to those students engaged in during course activities, but employed outside of course sessions and/or with individuals not in the course. For example, spacing out review of material harkens back to how the reformed class spaced out homework, and explaining concepts to family members is reminiscent of in-class peer discussion.

Student-Centered Strategies

Table S5: Constituent codes of the descriptive theme: *Student-centered strategies*

Code	Definition	Illustrative Quotes
breaking things down	Communicating complex material in approachable and/or accessible ways	"it was explained in simple words rather than big words. And if there were big words, they were explained" (R-1)<br><br>"she kind of got on our level where it's okay if you don't understand it because she made a point that's why she's here." (R-2)<br><br>"[supplemental instructor] was able […] to put it into kind of, I don't know how to--not chemistry language, [but] English. It made sense." (UA-1)<br><br>"[supplemental instructor] could really break things down." (UB)
building on prior concepts	Connecting course material to prior course content	"it's easier because each thing that we learn builds upon itself. So we learn things more efficiently" (R-1)<br><br>"everything builds on itself, hitting on [a topic] more later, it would start to make sense" (UB)
creating interest	Opportunities for students to develop interest in the course material	"if you don't have like a certain interest in chemistry. She'll get you interested." (R-1)<br><br>"[the course] changed my perception […] how it was shown in high school […] was more to get us more interested in it, where I feel like at the college level they expect like, you sit down and like pay attention more. But I feel like, it woulda been cooler if there was more like, stuff like that […] like baking and stuff" (UB)
emphasizing understanding	Focus on students conceptual meaning-making	"She was always patient and helping me, making sure that I understand this and not just that I memorize it, but actually understand this material and how to apply it to the tests and the problems and stuff." (R-1)<br><br>"[this class] helped me know that understanding things is really satisfying" (R-2)
explain why we're doing what we're doing	Transparency around the reason for course pedagogical practices	"one really great initiative was she would even just vocally say during class that a lot of her teaching styles and things that she did was based on research of what is found to work the best, and there were a lot of different things she did to try and make sure she was, I guess, hitting as many people as possible. And it just seemed she was a very effective teacher in that way." (R-1)<br><br>"she'd always tell us, 'you're also teaching yourself by teaching others because you have to make sure you know the material in order to teach'." (R-1)
Multiple ways of introducing content	Multiple avenues for students to engage with course content	"her posting videos really helped me understand the concept more after reading about it." (R-1)<br><br>"she was also really good at writing on the board and not just only showing slides" (R-2)

Real life connections	Opportunities for students to make connections between the course material and real world phenomena or experiences	"she would use examples from everyday life to try and explain stuff, which made it a lot easier" (R-2)<br><br>"I started to connect it to real life and real-world situations. And I started to kind of see it in everyday things and apply it to everyday situations. […] it gave me more meaning" (UA-2)<br><br>"with certain topics [supplemental instructor] was able to like relate it to like, the world itself so it would be easier to grasp that information" (UB)
spacing work out	Course structures that break the work into smaller pieces spread over time	"One of the things that she did that was very helpful personally for me was she kind of spread out the homework on an everyday basis rather than the end of the week." (R-1)<br><br>"we had one assignment due per week […] if she [had] split it up and given it due like one day 25 questions and then the next day 25, that would have helped me […] just split it up." (UA-1)

Instances of student-centered strategies were described in all focus groups, but we noted that participants from the unreformed sections (focus groups UA-1, UA-2, UB) nearly exclusively discussed such practices occurring in their interactions with the supplemental instructor in optional out-of-class meetings. Interestingly, Additionally, only students in the reformed section brought up the instructor's pedagogical transparency (code: "explain why we're doing what we're doing"). Interestingly, there were multiple instances where participants from unreformed sections provided ideas or suggestions for how the course and/or instructor might be better able to support their learning, many of which were the same or highly similar to student-centered practices highlighted by participants in focus groups R-1 and R-2 highlighted. For example, a participant from section UB commented on a desire for the course to attend more to creating interests, an a participant from section UA described a desire for the homework to be broken up with deadlines spaced out throughout the week (see illustrative quotes in Table 6).

Practice

Table S6: Constituent codes of the descriptive theme: *Practice*

Code	Definition	Illustrative Quotes
Going over explanations of answers	Reviewing reasoning behind answers to problems	"she would even make you explain why you think you're right or wrong just to make sure you understand the concept." (R-1)<br><br>"I feel like with chem, it should do some of [what my physics class does] [..] with the math questions, just going over those together and then probably at the end of the class, he will explain how to do each one properly." (UA-2)<br><br>"[supplemental instructor] does a really good job of like […] how to understand stuff. But like she also is really good at giving additional examples […] she goes through like every possible outcome" (UB)
in-class practice	Opportunities to apply and develop knowledge and skills during course sessions	"[she] would encourage us to kind of have partner talks. And I feel like that kind of helps because, one, it forces you to talk with someone in the class, but also just kind of have somebody that you can kind of piggy-back off. Because they might understand it in a way that you don't" (R-1)<br><br>"usually by the third or fourth time we were doing a practice thing in class, I felt like I was getting it." (R-2)<br><br>"I probably learned most when she was doing examples on the board […] the repetition because whenever she would do something on the board it was kind of like the same problem over and that really helps me." (UA-2)
out-of-class practice	Opportunities to apply and develop knowledge and skills outside of course sessions	"I went to her office hours multiple times in a week, and she even had special office hours, too. And so, she was really helpful during that time." (R-1)<br><br>"I use flash cards. Some people might think that's little prehistoric but I actually think that's it's helped for me" (R-2)<br><br>"I didn't do enough outside of class. […] I think I struggled because I didn't apply myself outside of the classroom" (UA-1)<br><br>" […] quizlet. Quizlet helped a lot." (UB)

Participants across all focus groups and course sections describe a variety of ways they engaged in practice. However, we noted that participants from the reformed course section (focus groups R-1, R-2) tended more often to discussed instances of practice during in-class activities or in out-of-class meetings with the instructor (e.g., office hours). In contrast,

participants from unreformed course sections (focus groups UA-1, UA-2, UB) tended more often to describe practice they engaged in with the supplemental instructor and/or independently. Additionally, participants from unreformed sections at times made comments about how things could or should have been different, which often highlighted the importance of practice in and out of class. As shown in Table 7, a UA-1 participant directly attributed difficulties they encountered to not having engaged in sufficient practice outside of class. Similarly, a UA-2 participant from Section UA-2 described a preference for the class to use in-class practice opportunities similar to those used in a different course. Notably, such practices were frequently used in the reformed section, and often highlighted by participants in focus groups R-1 and R-2.

Prior Experiences

The *Prior Experiences* theme reflects instances where participants discuss how experiences they had in the past impacted their attitude toward chemistry and the course. As shown in Table 8, it was common for participants to highlight prior experience with chemistry in high school, which could be described as positive or negative. However, many participants also described other aspects of their high school experience to inform their perception of chemistry and their attitudes about the course. For example, participants frequently mention impacts from their prior education or their experience with other science disciplines.

Table S7: Constituent codes of the descriptive theme: *Prior experiences*

Code	Definition	Illustrative Quotes
math skills	Perceived relationships between one’s mathematical training and/or skills, and their chemistry experience	“math is not my forte. No. Especially when it is like the long chain of conversions […] I did go over that in high school […] that one took me awhile to get back to again.” (R-2)<br><br>“I'm taking Calc I right now. I thought the math with chemistry was going to be easy, but then

		when we went to the conversions, I was just lost." (UA-1)<br><br>"depending on your major, if you're taking [this course], most of the time you're taking [time-intensive preparatory math course]. […] its like, you gotta get your math hours, and I'm just like—who has time to be doing all […] Like we going to have to split it between the two" (UB)
highschool → college	Descriptions of high school chemistry experiences impact college chemistry experiences	"I took chemistry in high school as well and I felt-- I liked it in high school so I felt like it was going to be good again. And it was" (R-1)<br><br>"I had a difficult time because I didn't take chemistry in high school." (R-2)<br><br>"Honestly, for me, I took physics in high school and physics helped me a lot for the laws part of chem. That was so easy for me because of physics." (UA-1)<br><br>"in high school, it was definitely easier so I kinda like came in like, oh—like, a little cocky, like oh this is super easy, especially after the first test […] it did get like really really challenging for me, so [this class] definitely lowered my confidence" (UB)

We did not notice differences in how focus group participants from different course sections described their prior preparation and/or experiences. In discussions from focus groups from all course sections, there tended to be a combination of positive, neutral, and negative prior experiences that participants described influencing their perceptions of chemistry and their feelings about enrolling in this chemistry course.
